# Biological Monitoring of Occupational Exposure to Metals in Electric Steel Foundry Workers and Its Contribution to 8-Oxo-7,8-Dihydro-2′-Deoxyguanosine Levels

**DOI:** 10.3390/ijerph17061811

**Published:** 2020-03-11

**Authors:** Laura Campo, Mariem Hanchi, Sabrina Sucato, Dario Consonni, Elisa Polledri, Luca Olgiati, Dalila Saidane-Mosbahi, Silvia Fustinoni

**Affiliations:** 1 Fondazione IRCCS Ca’ Granda Ospedale Maggiore Policlinico, 20122 Milan, Italy; dario.consonni@policlinico.mi.it (D.C.); luca.olgiati@policlinico.mi.it (L.O.); silvia.fustinoni@unimi.it (S.F.); 2 Laboratory of Analysis, Treatment and Recovery of Environmental Pollutants and Products, Faculty of Pharmacy, 5000 Monastir, Tunisia; meriamhanchi@yahoo.fr (M.H.); dalila.saidane@fphm.rnu.tn (D.S.-M.); 3 Department of Clinical Sciences and Community Health, Università degli Studi di Milano, 20122 Milan, Italy; sabrina.sucato@unimi.it (S.S.); elisa.polledri@unimi.it (E.P.)

**Keywords:** electric steel foundry, metals, urine, biological monitoring, occupational exposure, biological limit values, 8-oxo-7,8-dihydro-2′-deoxyguanosine, polycyclic aromatic hydrocarbons

## Abstract

In this study, the urinary concentrations of selected metals in workers from an electric steel foundry in Tunisia were assessed and compared with existing biological limit values and general population reference values. Moreover, the association between oxidative DNA damage, measured as urinary 8-oxo-7,8-dihydro-2’deoxyguanosine (8-oxodG) and co-exposure to metals and polycyclic aromatic hydrocarbons (PAHs) was evaluated. Urinary levels of 12 metals were determined by inductively coupled plasma-mass spectrometry (ICP-MS) in end-shift spot samples from 89 workers. The urinary levels of phenanthrene (U-PHE), as marker of exposure to PAHs, and 8-oxodG were also available. Median levels ranged from 0.4 µg/L (cobalt, Co, and thallium, Tl) to 895 µg/L (zinc, Zn). Only 1% of samples was above the biological limit values for Co, and up to 13.5% of samples were above limit values for Cd. From 3.4% (Co) to 72% (lead, Pb) of samples were above the reference values for the general population. Multiple linear regression models, showed that manganese (Mn), Zn, arsenic (As), barium (Ba), Tl, and Pb were significant predictors of 8-oxodG (0.012 ≤ *p* ≤ 0.048); U-PHE was also a significant predictor (0.003 ≤ *p* ≤ 0.059). The variance explained by models was low (0.11 ≤ R^2^ ≤ 0.17, *p* < 0.005), showing that metals and PAHs were minor contributors to 8-oxodG. Overall, the comparison with biological limit values showed that the study subjects were occupationally exposed to metals, with levels exceeding biological limit values only for Cd.

## 1. Introduction

The production of steel by recycling scrap using an electric arc furnace is a commonly used process. In Europe, it accounts for 41.8% of the total steel production [1]. The main processing operations of a typical steel foundry are scrap handling, metal melting, mold and core production, casting, and finishing. During the manufacturing process, steel foundry workers are potentially exposed to metal fumes (in particular Cr, Mn, Ni, and Cd), polycyclic aromatic hydrocarbons (PAHs), and other pollutants such as silica and quartz dust, and binder compounds (formaldehyde, resins, and oils) [2]. Metal fumes are formed by evaporation, condensation and oxidation of metals in air. Furnace tenders, smelters, casters, ladle-men, pourers and crane drivers are exposed to fumes from molten metal; fettlers are exposed to metal fumes and dusts from grinding, welding and flame-cutting operations. On the other side, PAHs mainly result from thermal decomposition of carbonaceous ingredients in foundry sand. Moreover, another source of PAHs is exhaust gases from engines, furnaces, and ovens [2].

Several cohort studies have shown an increased incidence of lung cancer [3] and bladder cancer [4] among steel foundry workers. Based on epidemiological evidences, the International Agency for Research on Cancer has classified the occupational exposure during iron and steel founding as carcinogenic to humans (Group 1) [2].

Data about metal emissions in air during steel production in the European Union, show that Zn is the metal with the highest emissions with levels up to 24,000 mg/t of liquid steel, followed by As (up to 14,000 mg/t), Pb, chromium (Cr), and nickel (Ni) (up to 2800 mg/t), copper (Cu) (up to 510 mg/t), and Cd (up to 148 mg/t) [1]. A study in Italy reported that iron was the principal constituent (78%) of the dust released as fine and ultrafine particulate matter through diffuse emissions by an electric steel foundry, followed by aluminum (Al), Zn, and Mn (5–7%), while other metals contributed less than 1% [5]. 

Only a few studies report personal or environmental exposure to metals in steel workers. The median personal concentration of respirable dust in an integrated-cycle steel foundry in Italy was below 0.1 µg/m^3^ for As and Cd, <2 µg/m^3^ for Cr, Ni, Cu, and Pb, and 7.3 µg/m^3^ for Mn [6]. In four nonferrous metal foundries in Brazil, environmental levels were up to 200 µg/m^3^ for Pb, up to 8 µg/m^3^ for Cd, up to 4.5 µg/m^3^ for Ni, and up to 1.6 µg/m^3^ for Mn [7]. The high variability of metal emission observed between foundries may originate from differences in the production process, input materials, type of steel produced, and plant characteristics.

While environmental monitoring is a useful tool to collect information about the metal concentration in the ambient air of a working place, biological monitoring provides an information about the internal dose of individuals, taking into account the intra-individual variability. The biological monitoring of metal exposure in steel workers has been very limited, with Ni, and Cd as the most investigated metals [6,7,8,9,10,11,12,13]. 

The exposure to metals is associated with toxic effects; various studies reported adverse health effects including neurotoxicity following exposure to As, Pb, and Mn [14], the development of cardiovascular diseases following exposure to Pb, Cd, and As [15], and other effects as kidney damage, endocrine disruption, and immunological effects [16]. Moreover, some metals such as As, Cd, Ni, and Cr (VI) are classified as carcinogenic to humans [2]. 

Metals, as well as PAHs, enhance oxidative stress by the generation of reactive oxygen species by various mechanisms. Oxygen free radicals can induce a variety of damage to DNA, such as the hydroxyl radical that binds DNA in the eight position of the guanine leading to its modification. The resulting 8-oxo-7,8-dihydro-2’-deoxyguanosine (8-oxodG) is considered a pre-mutagenic lesion and a biomarker of DNA oxidation [17,18]. 

In our previous study, we evaluated the exposure to PAHs in Tunisian electric steel foundry workers by measuring urinary level of 16 unmetabolized PAHs (U-PAHs), and eight hydroxylated metabolites of PAHs [19,20]. The analysis of a large panel of biomarkers allowed us to propose urinary unmetabolized phenanthrene (U-PHE) and 1-hydroxyphenanthrene as the most suitable biomarkers of PAH exposure [20]. In a successive work in the same population, we found a small, but significant contribution of urinary PAHs, particularly U-PHE, on 8-oxodG excretion [19].

In this study, we evaluated the exposure to metals in the same workers, by measuring a panel of 12 urinary metals (vanadium V, Cr, Mn, Co, Ni, Cu, Zn, total As, Cd, Ba, Tl, and Pb) and by comparing metal levels with existing biological limit values proposed by some international agencies and general population reference values. Moreover, the association between co-exposure to metals and PAHs on 8-oxodG excretion was investigated.

## 2. Materials and Methods 

### 2.1. Subjects and Urine Collection 

The sampling took place in August 2013 at an electric steel foundry in Tunisia previously described [20]. A self-administered questionnaire was used to obtain subject’s personal information, including personal characteristics, smoking habits, and job title.

Workers were classified into six groups based on their job title and workshop (department): (1) steel smelters workers (SSW, N = 30), operating in the steel smelting workshop in which scrap and ferrous alloys are first melted by an electric arc furnace (EAF), and then conducted to a continuous casting process in the same area; main job tasks are smelters, molders, and casters. (2) Rolling mills (N = 34) and cable fiber workers (N = 2) (RM-CF, N = 36), operating in the rolling mill workshop where metal is heated and then passed in a forming process to give it the final shape, in particular cable fiber workers produce fine cables by heating the rolled product; main job tasks are rolling-mill operators, and machine operators. (3) Galvanization workers (GALV, N = 7), operating in the galvanization workshop, where the products are immersed in a hot zinc bath to coat the steel and prevent it from rusting. (4) Engine maintenance workers (MAN, N = 12), repairing and maintaining engines using mainly welding and flame-cutting operations. (5) Measure and quality control workers (QC, N = 4), operating in a separate department where adequate quality of products is assessed by measuring, examining, and testing the characteristics of the products. (6) Individuals (ADM, N = 4,) with job tasks non involving direct contact with metals (security, material transport inside the factory, and administrative job tasks).

Urine spot samples from each subject were collected at the end of an 8-h work shift. Samples were blind coded, shipped to the laboratory in dry ice, and frozen at −20 °C until analyses. All participants gave their informed consent about their inclusion in the study. The study was approved by the ethics commission of the University of Medicine of Monastir (Tunisia).

### 2.2. Measurement of Urinary Metals 

Before analysis, urine samples were thawed at room temperature for 2 h. Each sample was mixed and heated at 37 °C for 30 min to dissolve the sediment. An aliquot of 600 µL of urine was transferred into a 10 mL polyethylene tube and added with 2.4 mL of nitric acid 0.05% *v*/*v*, prepared by dilution of ultrapure nitric acid (69% TraceSelect, Fluka, France), and containing 45Sc, 89Y, and 111In at a concentration of 7.5 µg/L as internal standards (Inorganic Ventures, Inc., Lakewood, NJ, USA). All solutions were prepared using Milli-Q^®^ ultrapure water (conductivity 0.056 µS/cm) (Merck, Darmstadt, Germany). 

Samples were analyzed by an inductively coupled plasma-mass spectrometer (ICP-MS) X Series II (Thermo Electron Corporation, Rodano, Italy) equipped with standard nickel cones, torch, and quartz impact bean spray chamber and interfaced to an auto sampler ASX-100 (Teledyne CETAC Technologies, Omaha, NE, USA). The instrument was operated with standard mode for Pb, Zn, Ba, Tl, and Cd and collision cell mode (CCT-Ked) for Mn, Ni, Cu, V, As, Co, and Cr. The typical standard mode conditions were as follows: extraction voltage −100 V, focus voltage 8.5 V, nebulizer gas flow rate 0.90 L/min and dwell time 50 ms for each element. For each sample, three replicates were run. In CCT-Ked, a cell gas flow of 3.5 mL/min of 8% *v*/*v* hydrogen in helium was used to reduce interferences.

Before each analytical sequence, the instrument was calibrated using the tune solution obtained by dilution 1:50 of the multi-element solution Tune A (containing Ba, Be, Ce, Co, In, Li, Ni, Pb, each at 10 mg/mL in 5% HNO_3_) (Analytika, Prague, Czech Republic).

The calibration curves were in the range 0.1–50 µg/L for all analytes, but Zn, for which a calibration curve in the range 50–1000 µg/L was used. The calibration solutions were obtained by dilution of the multi-element standard stock solution 71A, containing all elements analyzed at 10 mg/mL (Inorganic Ventures, Inc., Lakewood, NJ, USA), with nitric acid 0.05% *v*/*v* containing 45Sc, 89Y, 111In, at 7.5 µg/L as internal standards. The calibration curves for each metal were linear with correlation coefficient ≥0.999. The limits of quantification (LOQs), calculated as ten times the standard deviation of the blank signal, were as follows: V 0.02, Cr 0.05, Mn 0.06, Co 0.02, Ni 0.18, Cu 0.03, Zn 0.71, As 0.5, Cd 0.03, Ba 0.03, Tl 0.02, and Pb 0.07 µg/L. Internal quality assurance was performed using two quality controls (QCs) for metals in urine: Lyphocheck Urine Metals Control, Level-1 (Bio-Rad Laboratories, USA), and Seronorm^®^ Level-1 (Sero AS, Billingstad, Norway). Before analysis, these controls were reconstituted according to the manufacturer’s instruction. For quality controls, precision, as the variation coefficient, was <10% and the accuracy was 85–115% of the theoretical values. 

### 2.3. Measurement of U-PHE, 8-oxodG and Cotinine

The U-PHE was analyzed, together with other 15 U-PAHs, by solid phase microextraction coupled with gas chromatography-triple quadrupole tandem mass spectrometry, as previously described [20,21].

Urinary 8-oxodG and urinary cotinine (U-COT) were analyzed simultaneously by liquid chromatography coupled with triple quadrupole mass spectrometry, as previously described [19,22]. U-COT was measured to characterize each subject’s smoking status. Subjects with U-COT ≥ 30 µg/L were classified as smokers [23]. 

Creatinine (cr) was determined photometrically as picrate using Jaffe’s colorimetric method [24].

### 2.4. Statistical Analysis

Statistical analyses were carried out using the SPSS package for Windows (version 25.0; SPSS Statistics, IBM, Italy) and the Stata 13 package (version 2013; Stata Corp LP, College Station, TX, USA). A value corresponding to one-half of the quantification limit was assigned to measurements below analytical quantification. Data on urinary biomarkers were decimal log-transformed to ensure normal distribution. Student’s *t*-test was applied to compare two independent groups (i.e., smokers vs. nonsmokers), analysis of variance (ANOVA) with Bonferroni correction was applied to compare more than two independent groups (i.e., job titles). Pearson’s correlations were used to measure the associations between quantitative variables.

Multiple linear regression models were used to evaluate the effect of smoking status (log_10_ U-COT, µg/L), urinary creatinine (log_10_, g/L), age (years), and body mass index (BMI, kg/m^2^) as independent variables on the urinary levels of each metal (log_10_ metal, µg/L) (dependent variable). The final model was:

Urinary metal = intercept + β1 × U-COT + β2 × creatinine + β3 × age + β4 × BMI

Two different multiple linear regression models, Model A and Model B, were used to study the association between urinary 8-oxodG (dependent variable), and metal doses or metal and PAH co-exposure (independent variables). Model A evaluated the effect of the urinary level of each metal (log_10_ metal, µg/L), smoking status (log_10_ U-COT, μg l/L), urinary creatinine (log_10_, g/L), age (years), and BMI (kg/m^2^) on the 8-oxodG levels (log_10_, μg/L). Each regression analysis was run separately for each metal. The final model A was:

8-oxodG = intercept + β1 × urinary metal + β2 × creatinine + β3 × U-COT + β4 × age + β5 × BMI

Model B evaluated the effect of the urinary level of each metal (log_10_ metal, µg/L), PAH exposure (log_10_ U-PHE ng/L), smoking status (log_10_ U-COT, μg /L), urinary creatinine (log_10_, g/L), age (years), and BMI (kg/m^2^) on the 8-oxodG levels (log_10_, μg/L). Each regression analysis was run separately for each metal and U-PHE together. The final Model B was:

8-oxodG = intercept +β1 × urinary metal + β2 × U-PHE + β3 × creatinine + β4 × U-COT + β5 × age + β6 × BMI

For each model, the regression slopes of the resulting linear equations were anti-log10 converted to obtain the geometric mean ratio (GMR). The (GMR−1) × 100 gives the percentage increase or decrease of 8-oxodG for each 10-fold increase of each urinary metal or U-PHE concentration. 

## 3. Results

### 3.1. Study Population

Main characteristics of the study population were previously reported [20]. Of the 93 male workers participating to the study, urine samples for metal analysis were available for 89 individuals. In these subjects, age ranged from 25 to 59 years (mean 47 years) and BMI ranged from 20 to 34 (mean 27 kg/m^2^). Based on U-COT excretion, 51 (57%) subjects were classified as smokers.

### 3.2. Urinary Metal Levels

Table 1 shows the results of metal analyses and statistical comparisons among job titles. Results are shown for all workers and for workers stratified by job titles. In all subjects, median levels ranged from 0.4 µg/L (Co and Tl) to 895 µg/L (Zn) (Table 1). All metals were above the LOQ in all samples, but one sample for Cd. 

Median levels were significantly (*p* < 0.05) or marginally different (0.05 < *p* < 0.1) among job titles for all metals, except Cr and Mn. In particular, the SSW group had the lowest levels of Co, Cu, Zn, As, Cd, Ba, Tl, and Pb in comparison with the other groups. On the contrary, no significant differences were found among the other job titles (Table 1).

Median levels were significantly different between smokers and non-smokers only for V (0.88 vs. 0.59 µg/L in non-smokers and smokers, *p* = 0.001) and Tl (0.3 vs. 0.4 µg/L in non-smokers and smokers, *p* = 0.022).

Urinary metals were generally correlated within each other’s: partial Pearson’s r correlation, when significant, ranged from 0.218 (As vs. Cr, *p* = 0.040) to 0.850. The highest correlations were found for Cd vs. Pb (r = 0.850, *p* < 0.0001), Cd vs. Tl (r = 0.838, *p* < 0.001), and Tl vs. Pb (r = 0.836, *p* < 0.001) (data not shown). 

### 3.3. U-PHE and 8-oxodG Levels

The results of the analysis of U-PHE, together with the analysis of other U-PAHs and hydroxylated metabolites, and the results of the analysis of 8-oxodG were previously reported [19,20] and here are only summarized. Briefly, in all subjects, U-PHE median level (5th–95th percentile) was 18.9 (7.1–74.5) ng/L, while 8-oxodG median level (5th–95th percentile) was 3.19 (0.84–14.94) μg/L [1.85 (0.46–9.05) μg/g creatinine]. 

### 3.4. Correlations between Urinary Metals, U-PHE, and 8-oxodG

Significant, or marginally significant, linear correlations were found between 8-oxodG and the following metals: Mn (r = 0.354, *p* = 0.001), Tl (r = 0.321, *p* = 0.002), Zn (r = 0.309, *p* = 0.003), As (r = 0.309, *p* = 0.003), Pb (r = 0.283, *p* = 0.007), Co (r = 0.239, *p* = 0.024), Ba (r=0.213, *p* = 0.045), Cr (r = 0.213, *p* = 0.045), and Cd (r = 0.194, *p* = 0.071). 

Significant correlations were found between U-PHE and some metals, specifically Mn (r = 0.454, *p* ≤ 0.001), and Zn (r= 0.225, *p* = 0.034). 

A significant linear correlation between U-PHE and 8-oxodG was found (r = 0.327, *p* < 0.001) [18].

### 3.5. Multiple Regression Analysis

The multiple linear regression model predicting urinary metals as a function of age, BMI, smoking habit, and creatinine showed that most metals were significantly associated with age (Co, Cu, Zn, As, Cd, Tl, and Pb, 0.001 < *p* ≤ 0.042) and creatinine (Cr, Mn, Co, Ni, Cu, Zn, As, Tl, and Pb, 0.003 < *p* ≤ 0.075), while smoking habit and BMI were never significant predictive factors.

Table 2 shows the results of multiple regression analyses (Model A and Model B). In model A, studying the association between 8-oxodG and the exposure to each metal, the coefficient of determination (R^2^) ranged from 0.03 for the model with Ni as independent variable, to 0.12 for the model with Mn as independent variable (0.007 ≤ *p* ≤ 0.162) (Table 2). Mn, Zn, As, Tl, and Pb were significant predictors of 8-oxodG (0.003 ≤ *p* ≤ 0.027), while Co and Ba were marginally associated to 8-oxodG (0.05 ≤ *p* ≤ 0.10). The calculated increase of 8-oxodG excretion ranged from 41% (Ba) to 88% (Mn) for each 10-fold increase of the metal excretion.

In Model B, studying the association between 8-oxodG and the co-exposure to each metal and PAHs, the coefficient of determination (R^2^) ranged from 0.11 for the models with V, Ni or Cu and U-PHE as independent variables, to 0.17 for the model with Tl and U-PHE as independent variables (0.002 ≤ *p* ≤ 0.017) (Table 2). Mn, Zn, As, Ba, Tl, and Pb were significant predictors of 8-oxodG (0.012 ≤ *p* ≤ 0.048). The calculated increase of 8-oxodG excretion ranged from 45% (Ba) to 74% (Tl) for each 10-fold increase in metal excretion. U-PHE was always a significant predictor of 8-oxodG (0.003 ≤ *p* ≤ 0.059), with increases in the 90%–158% range for each 10-fold increase in U-PHE excretion.

Creatinine, U-COT, age, and BMI were not significant predictive factors of 8-oxdG in any models (results not shown).

## 4. Discussion

In this paper, metal exposure in Tunisian steel foundry workers was assessed by measuring 12 urinary metals and by comparing metal levels with existing occupational limit values and general population reference values. Moreover, the role of the exposure to metals in determining an oxidative stress status was investigated, also in relationship with PAH co-exposure. As far as we know, this is the first time that the occupational exposure to metals has been evaluated by measuring a wide range of urinary metals in Tunisia. 

With the aim of protecting workers’ health, reference values for the biological monitoring of occupational exposure were proposed by some international agencies (summarized in Table 3). For the urinary metals here studied, the American Conference of Governmental Industrial Hygienists (ACGIH) recommends a biological exposure index (BEI) for Cr, Co, and Cd [25]. The Commission for the Investigation of Health Hazards of Chemical Compounds in the Work Area, MAK Commission (issued from the German Research Association DFG—Deutsche Forschung Gemeinschaft), provides exposure equivalents for carcinogenic substances (EKA) for V, Co, and Ni, a biological guidance value (BLW) for Co, and biological reference values for workplace substances (BAR) for Cr, Co, Ni, Cd, and Ba [26]. It is worth underlining that the EKA values are “exposure equivalents”, that is the concentrations of a substance or its metabolites in biological material which is known to correspond to the concentration of the substance in the workplace air. From these relationships, the body burden which results from uptake of the substance exclusively by inhalation may be determined. So, the biological value is given together with the corresponding air level [26]. The Risk Assessment Committee (RAC) of the European Chemicals Agency (ECHA) provides a biological limit values (BLV) for Cd [27] (Table 3). 

In comparison with these indexes, one sample for Co was above the ACGIH BEI and above the 30 µg/L DFG EKA (corresponding to an environmental exposure above 0.050 mg/m^3^) but below the DFG BLW value. For Cd, 3.4% and 13.5% of samples were above the ACGIH BEI and the ECHA BLV, respectively. ECHA has provided a limit value lower than ACGIH (2 vs. 5 µg/g creatinine) and it has classified this metal as a carcinogen Group C, that is a genotoxic carcinogen for which a mode of action-based threshold can be identified and a health-based OEL is proposed [28]. No sample was above the ACGIH BEI for Cr and no sample was above the DFG EKA for V and Ni (Table 3).

Results of biological monitoring of urinary metals in Tunisia are available only for Pb. The levels of Pb found in study workers were much lower than those in a small control group of 20 individuals (5.0 vs. 38 µg/L), but it should be noted that the reported values were surprisingly high [29]. The metals levels were then compared with the reference values for the Italian general population provided by the Italian Society of Reference Values (SIVR) [30], and with the DFG BAR (Table 3). From 3.4% (Co) to 72% (Pb) of samples were above the reference values for the Italian population. In particular, 63, 19, 14, and 69% of samples were above the reference values for Cr, Ni, As, and Cd, respectively. In comparison with the BAR values, from 7 (Co) to 73% (Cd) of samples were above the BAR values for Co, Ba, Ni, Cr, and Cd (Table 3).

Overall, these results show that the study workers were occupationally exposed to metals, but the exposure was within biological limit values for most metals. Only for Cd, up to 13.5% of workers exceeded the biological limit values, in particular the limit proposed by ECHA.

Table 4 shows a comparison with previous studies where biological monitoring of steel workers has been performed using urinary metals as biomarkers of exposure. The comparison with previous studies is difficult because of the variability of the production process between foundries and the lack of process description in many studies. Moreover, most studies investigated a limited numbers of metals, mostly Ni, Cd, and Cr, while spurious studies were found for the other metals (Table 4). The levels of Ni, Cd, Cr, Pb, and Cu were 2–5 fold higher than levels found in other steel foundries [6,7,8,13], or in a copper foundry [9]. Levels of Zn were two fold higher than those reported by the only study reporting urinary Zn in steel foundry workers [6]. 

For As, it is worth to highlight that we measured total arsenic in urine (that is the sum of inorganic and organic As), while other studies measured inorganic arsenic [6,8,9]. This is the reason why in this study 2–5 fold higher As levels were found. Arsenic can be found in considerable amount in food and drinking water: a daily intake of total As from food and beverages is generally in the range of 20–300 μg/day [31]. However, the arsenic in food is mainly associated with the presence of arsenobetaine and arsenocholine that are considered non-toxic [31,32]. Regional differences are seen in the daily intake of total arsenic through food, mainly attributable to the type and the quantity of food consumed. In particular, seafood is known to give a major contribution to the ingestion of arsenic [32]. As the investigated plant is located in the Northern Tunisia, close to the Mediterranean Sea, it is probable that seafood is a relevant contributor to the diet of the investigated individuals. Unfortunately, this cannot be verified, as data about worker’s diet were not collected. 

Levels of Mn were comparable to those found in an integrated steel foundry [6] and in a non-ferrous metal smelting plant [7], but four-fold lower than those from a steel mill production plant [12]. Levels of Tl and Ba were comparable to the only values found in the literature about urinary Tl [10] and Ba [6] in steel foundry workers and to that reported for thallium workers [33].

Levels of Co were much lower (8–20 fold) than levels found in foundry workers from a steel mill production plant in Pakistan [12] and a steel plant in Taiwan [11] and comparable to those of an Italian integrated steel foundry [6]. As far as we know, no studies reported V urinary levels in foundry workers. Levels found in this study were much higher than those reported for welders (median 0.08 µg/g cr) [34]. In summary, the comparison with previous studies shows that the levels of Ni, Cd, Cr, Pb, Cu, and Zn were higher, while the levels of Mn, Tl, Ba, and Co were comparable or lower than those found in other steel foundries. 

Considering the different job titles, the SSW had the lowest levels for most metals. One reason of this unexpected result could be the workshop design, which has openings on both the opposite sides. This may favor the ventilation and thus reduce the exposure of these workers. On the contrary, no differences in metal levels were found among the other job titles, which worked in closed departments.

The regression analysis predicting the urinary levels of metals showed that age was a significant determinant for Co, Cu, Zn, As, Cd, Tl, and Pb. For Cd and Pb, this is in agreement with the known tendency for these metals to accumulate in the body [35,36]. Otherwise, the smoking habit was not a determinant factor for any metal. Tobacco smoke is a known significant source of cadmium: it has been estimated that tobacco smokers are exposed to 1.7 μg cadmium per cigarette [37]. Notwithstanding the effect of cigarette smoke was expected, we did not find such an association, this may be explained by the high Cd levels reasonably due to occupational exposure.

In a previous study, we evaluated 8-oxodG levels, used as a marker of oxidative damage, and its association with PAH exposure [19]. Results showed that 8-oxodG levels were in the range of the general population for healthy Italian individuals (5th–95th percentile: 1.75–10.2 µg/g creatinine) [38]. Moreover, although urinary PAHs were associated to 8-oxodG, they were only minor contributors to 8-oxodG excretion [19]. That finding made us to suppose that 8-oxodG was possibly affected by the metal exposure and then to study the co-exposure to metals and PAHs and the possible additive effect on 8-oxodG excretion. In this study, positive correlations between most of the investigated urinary metals (Mn, Tl, Zn, As, Pb, Co, Ba, Cr, and Cd) and 8-oxodG were found, with Pearson’s *r* in the 0.194–0.345 range. The multiple regression model, studying the association between 8-oxodG and urinary metals and corrected for confounding factors (model A), confirmed the positive association for all metals, but for Cr and Cd. However, the variance explained by this model was low (3–12%), showing that metal exposure was not the major contributor to 8-oxodG (Table 2). This result is partially in accordance to what previously reported for coke-oven workers in China, showing that As and Ni were weak predictors of 8-oxodG, while no association was found for Cd, Cr, and Pb [39]. Pb and Cd resulted significant predictor of 8-oxodG also in steel-iron mining and smelting workers in China, while no association was found for Cu and Ni [13]. Studying the association between 8-oxodG and the co-exposure to metals and PAHs corrected by confounding factors (model B), positive associations were found for the same metals, with the exception of Co, and U-PHE was a positive significant predictor too. The variances explained by model B (11–17%) were higher than model A, suggesting that the co-exposure to metals and PAHs enhances 8-oxodG excretion (Table 2). Similar findings showed an additive interaction effect of As, Cd, Cr, Ni, Pb, and PAH co-exposure on 8-oxodG excretion in coke oven workers [39], while a study among traffic conductors found an additive effect of co-exposure to Cd and PAH (but not Ni, As, and Pb) on 8-oxodG [40]. Overall, our results, together with previous studies, seem to point to the existence of an addictive interaction between metal and PAH exposure on 8-oxodG levels, even if this effect is weak and involving different metals. Discrepancies among studies may depend on differences in both PAH and metal exposure levels. The variance explained by models remains low (up to 17%) suggesting that higher doses of PAHs and/or metals are required to induce oxidative damage to DNA. Moreover, the comparison of the relative contribution of each metal and PAH exposure on 8-oxodG, show a major role played by U-PHE, with 8-oxodG increase due mainly to U-PHE than to metals.

## 5. Conclusions

In conclusion, the comparison with existing occupational limit values and with reference value for the general population shows that the study workers were occupationally exposed to metals, but the their exposure was within the biological limit values for most metals. The multiple regression models studying the association between 8-oxodG and the co-exposure to metals and PAHs explained only a minor part of the observed variance. This indicates that neither the exposure to metals nor the co-exposure to metals and PAHs are major determinants of 8-oxodG excretion.

## Figures and Tables

**Table 1:** Urinary metal levels in all study subjects and in subjects stratified by job titles. Median (5^th^-95^th^) is shown for each metal. The results of the statistical comparison among job title is also reported.

Metal	All Workers n = 89	*Workers Stratified by Job Titles*						*P ^a^*	*p ^b^*
		RM-CF n = 33	SSW n = 29	MAN n = 12	GALV n = 7	QC n = 4	ADM n = 4		
	Median (5th–95th)								
**V (µg/L)**	0.85 (0.05–2.40)	1.20 (0.6–2.87)	0.67 (0.21–2.78)	0.58 (0.03–1.97)	0.10 (0.07–2.27)	1.33 (0.05–1.49)	0.61 (0.05–1.78)	0.044	
**Cr (µg/L)**	0.75 (0.18–5.51)	0.84 (0.16–5.51)	0.57 (0.18–3.22)	0.50 (0.07–1.82)	1.00 (0.06–1.76)	2.30 (0.18–7.01)	0.84 (0.25–1.95)	0.198	
**Mn (µg/L)**	0.6 (0.1–4.1)	0.6 (0.1–2.8)	0.5 (0.1–3.3)	0.5 (0.2–5.0)	1.1 (0.2–1.7)	1.6 (0.1–4.1)	2.4 (0.3–10.3)	0.301	
**Co (µg/L)**	0.4 (0.1–1.9)	0.7 (0.2–4.2)	0.2 (0.0–0.9)	0.5 (0.1–1.2)	0.6 (0.0–1.1)	0.7 (0.2–2.2)	0.7 (0.3–32.0)	0.001	SSW vs. MAN = 0.002 SSW vs. ADM = 0.010
**Ni (µg/L)**	2.4 (0.5–6.3)	3.0 (0.7–6.1)	2.1 (0.6–8.5)	2.0 (0.5–5.4)	1.5 (0.3–3.2)	3.1 (0.8–6.2)	3.3 (1.4–7.5)	0.077	
**Cu (µg/L)**	25 (5–54)	29 (10–50)	11 (5–47)	23 (4–62)	25 (2–37)	36 (7–51)	41 (16–73)	0.007	SSW vs. RM-CF = 0.010
**Zn (µg/L)**	895 (137–3191)	1054 (272–3185)	390 (74–1270)	1153 (260–3986)	1533 (24–2750)	765 (302–3618)	3848 (862–7179)	<0.001	SSW vs. MAN = 0.060 SSW vs. ADM = 0.002 SSW vs. RM-CF = 0.003
**total As (µg/L)**	29 (3–156)	26 (4–1053)	30 (2–192)	51 (9–155)	48 (2–106)	27 (9–139)	41 (13–108)	0.028	SSW vs. MAN = 0.026
**Cd (µg/L)**	1.43 (0.10–4.57)	1.83 (0.59–6.39)	0.50 (<0.03–2.60)	1.85 (0.34–2.80)	1.88 (0.20–2.78)	2.15 (0.55–5.08)	3.23 (1.45–5.83)	<0.001	SSW vs. MAN = 0.006 SSW vs. RM-CF < 0.001 SSW vs. MAN = 0.006
**Ba (µg/L)**	3.5 (0.3–17.0)	5.4(1.6–17.0)	1.6 (0.2–6.5)	5.7 (1.0–16.7)	3.5 (0.2–31.7)	6.3 (2.1–10.3)	4.1 (2.0–13.6)	<0.001	SSW vs. MAN = 0.009 SSW vs. RM-CF < 0.001
**Tl (µg/L)**	0.4 (0.04–1.1)	0.5 (0.1–1.5)	0.1 (0.0–0.4)	0.6 (0.2–0.9)	0.5 (0.1–1.0)	0.7 (0.1–1.0)	0.6 (0.4–1.2)	<0.001	SSW vs. MAN < 0.001 SSW vs. GALV = 0.022 SSW vs. RM-CF < 0.001 SSW vs. ADM = 0.004 SSW vs. QC = 0.053
**Pb (µg/L)**	5.0 (0.8–19.0)	5.4 (2.0–19.1)	2.1 (0.4–11.0)	9.3 (3.0–19.0)	7.4 (0.6–25.6)	6.8 (2.9–12.5)	8.2 (50–29.2)	<0.001	SSW vs. MAN < 0.001 SSW vs. RM-CF < 0.001 SSW vs. ADM = 0.007

n = number of samples; ^a^ = *p* values represent significance of ANOVA for comparison among job titles; ^b^ = *p* values represent significance of ANOVA post hoc multiple comparison with Bonferroni correction (only *p* values ≤ 0.1 are shown).

**Table 2:** Results of multiple linear regression analyses for predicting urinary levels of 8-oxodG as a function of metal exposure only (Model A) and as a function of co-exposure to metal and polycyclic aromatic hydrocarbons (PAHs), (Model B). Independent variables were the urinary levels of each single urinary metal in Model A, and the urinary levels of each single urinary metal plus urinary phenanthrene (U-PHE) in Model B.

Model A			Model B		
Independent Variables	GMR (95% CI); *p* Value ^a^	R^2^_adj_^b^; P ^c^	Independent Variables	GMR (95% CI)_;_ *p* Value^a^	R^2^_adj_^b^; P ^c^
**V**	0.82 (0.58–1.18); 0.282	0.04; 0.155	**V**	0.87 (0.62–1.23); 0.430	0.11; 0.017
			**U-PHE**	2.42 (1.28–4.58); 0.007	
**Cr**	1.44 (0.93–2.21); 0.100	0.05; 0.087	**Cr**	1.40 (0.46–2.13); 0.110	0.13; 0.008
			**U-PHE**	2.44 (1.30–4.57); 0.006	
**Mn**	1.88 (1.25–2.85); 0.003	0.12; 0.007	**Mn**	1.61 (1.04–2.50); 0.033	0.15; 0.004
			**U-PHE**	1.90 (0.97–3.70); 0.059	
**Co**	1.45 (0.95–2.21); 0.085	0.06; 0.079	**Co**	1.37 (0.91–2.07); 0.128	0.13; 0.009
			**U-PHE**	2.38 (1.27–4.47); 0.008	
**Ni**	1.36 (0.75–2.43); 0.306	0.03; 0.162	**Ni**	1.31 (0.74–2.30); 0.348	0.11; 0.016
			**U-PHE**	2.46 (1.30–4.63); 0.006	
**Cu**	1.41 (0.80–2.49); 0.234	0.04; 0.141	**Cu**	1.32 (1.32–2.28); 0.323	0.11; 0.015
			**U-PHE**	2.42 (1.28–4.57); 0.007	
**Zn**	1.79 (1.16–2.75); 0.009	0.10; 0.017	**Zn**	1.61 (1.05–2.48); 0.029	0.15; 0.003
			**U-PHE**	2.17 (1.16–4.08); 0.016	
**total As**	1.52 (1.08–2.13); 0.016	0.09; 0.025	**As**	1.47 (1.84–2.04); 0.021	0.16; 0.003
			**U-PHE**	2.36 (1.28–4.38); 0.007	
**Cd**	1.38 (0.92–2.07); 0.114	0.06; 0.080	**Cd**	1.36 (1.84–2.04); 0.121	0.12; 0.010
			**U-PHE**	2.35 (1.28–4.38); 0.009	
**Ba**	1.41 (0.96–2.07); 0.081	0.06; 0.077	**Ba**	1.45 (1.00–2.09); 0.048	0.14; 0.005
			**U-PHE**	2.58 (1.38–4.80); 0.003	
**Tl**	1.82 (1.17–2.84); 0.008	0.10; 0.016	**Tl**	1.74 (1.13–2.67); 0.012	0.17; 0.002
			**U-PHE**	2.33 (1.26–4.31); 0.008	
**Pb**	1.73 (1.07–2.81); 0.027	0.08; 0.037	**Pb**	1.62 (1.01–2.59); 0.046	0.14; 0.004
			**U-PHE**	2.32 (1.24–4.34); 0.009	

GMR = geometric mean ratio. ^a^ = *p* values represent significance of each predictive variable. ^b^ = *R*^2^_adj_ values represent the adjusted coefficient of determination for the linear regression model. ^c^ = *P* values represent the significance of the linear regression model.

**Table 3:** Occupational limit values and reference values for the general population for urinary metals proposed by different organizations. The percentage of samples of study workers exceeding the biological values is shown.

Metal	Biomarker	Organization	Sampling Time	Biological Value	Value		% of Samples Above the Biological Value
**Vanadium** and its inorganic compounds including vanadium pentaoxide	**urinary V**	DFG	End of exposure, for long-term exposures: at the end of the shift after several shifts	EKA	Air (mg/m^3^)	Biomarker (µg/g creatinine)	
					0.025	35	0
					0.050	70	0
					0.100	140	0
		SIVR	-	SIVR	0.0250–0.855 µg/L		48
**Chromium** (VI), water soluble fume	**urinary Cr**	ACGIH	End of shift at end of workweek	BEI	25 µg/L		0
			Increase during shift	BEI	10 µg/L		2
**Chromium** and its compounds		DFG	End of shift	BAR	0.6 µg/L		63
		SIVR	-	SIVR	0.050–0.60 µg/L		63
**Manganese**	**urinary Mn**	SIVR	-	SIVR	0.040–1.5 µg/L		15
**Cobalt** and its compounds	**urinary Co**	ACGIH	End of shift at end of workweek	BEI	15 µg/L		1
		DFG	End of exposure, for long term exposure	EKA	Air (mg/m^3^)	Biomarker (µg/L)	
					0.010	6	0
					0.025	15	0
					0.050	30	1
					0.100	60	0
					0.500	300	0
				BLW	35		0
				BAR	1.5		7
		SIVR	-	SIVR	0.077–2.2		3
**Nickel** (nickel metal, oxide, carbonate, sulfide, sulfidic ores)	**urinary Ni**	DFG	For long-term exposures: at the end of the shift after several shifts	EKA	Air (mg/m^3^)	Biomarker (µg/L)	
					0.10	15	0
					0.30	30	0
**Nickel** and its compounds					0.50	45	0
			For long-term exposures: at the end of the shift after several shifts	BAR	3 µg/L		42
		SIVR	-	SIVR	0.372–4.44 µg/L		19
**Copper** and its inorganic compounds	**urinary Cu**	DFG	-	BAT	NA		
			-	BAR	NA		
		SIVR	-	SIVR	5.01–24.0 µg/L		52
**Zinc**	**urinary Zn**	SIVR	-	LVR	ND-1048 µg/L		40
**Cadmium** and its inorganic compounds	**urinary Cd**	ACGIH	Not critical	BEI	5 µg/g creatinine		3
		ECHA	Not critical	BLV	2 µg/g creatinine		14
		DFG	Not fixed	BLW	NA		
			Not fixed	BAR (NS)	0.8 µg/L		73
		SIVR	-	SIVR	0.100–0.900 µg/L		69
**Barium** compounds, soluble	**urinary Ba**	DFG	End of shift/ for long-term exposures: at the end of the shift after several shifts	BAR	10 µg/L		14
		SIVR	-	LVR	ND-6.97 µg/L		24
**Thallium**	**urinary Tl**	SIVR	-	SIVR	0.0600–0.759 µg/L		15
**Lead**	**urinary Pb**	SIVR	-	SIVR	1.170–2.94 µg/L		72

ACGIH = American Conference for Governmental industrial Hygiene; DFG = German Research Association (Deutsche Forschung Gemeinschaft); ECHA = European Chemicals Agency; SIVR = Italian Society for Reference Value. BAR = biological reference values for workplace substances; BAT = biological tolerance values (Biologische Arbeitsstoff-Toleranzwerte); BEI = Biological Exposure Index; BLV = biological limit values; BLW = biological guidance value (Biologische Leit-Werte); EKA = exposure equivalents for carcinogenic substances (Expositionsäquivalente für krebserzeugende Arbeitsstoffe); LVR = literature reference value; ND = not determined; NS = nonsmokers; NA = insufficient data for the derivation of a value.

**Table 4:** Literature summary of biological monitoring studies reporting urinary metal levels in foundry workers. The production process and the analytical assay used to quantify metals in urine is also shown.

Authors, Year *Country*	Production Process (Analytical Method)	N	V µg/L	Cr µg/L	Mn µg/L	Co µg/L	Ni µg/L	Cu µg/L	Zn µg/L	As µg/L	Cd µg/L	Ba µg/L	Tl µg/L	Pb µg/L
This Study, 2019 *Tunisia*	Electric steel foundry (ICP-MS)	89	0.85 (0.05–2.40) ^a^	0.75 (0.18–5.51) ^a^	0.6 (0.1–4.1) ^a^	0.4 (0.1–1.9) ^a^	2.4 (0.5–6.3) ^a^	25 (5–54) ^a^	895 (137–3191) ^a^	29 (3–156) ^a^ (total As)	1.43 (0.10–4.57) ^a^	3.5 (0.3–17.0) ^a^	0.4 (0.04–1.1) ^a^	5.0 (0.8–19.0) ^a^
Apostoli et al., 1988 *Italy* [10]	Cast iron foundry cupola furnace (X-ray fluorescence spectrometry)	21	-										0.33 (0.06–1.04) ^b^	
Horng et al., 2003 *Taiwan* [11]	Steel production plant (Differential pulse Stripping voltammetry)	63	-	-	-	8.18 (3.06–23.30) ^b^	33.10 (13.90–78.90) ^b^	-	-	-	9.52 (3.19–22.07) ^b^	-	-	53.50 (28.90–85.60) ^b^
Afridi et al., 2009 *Pakistan* [12]	Steel mill Production (GFAAS)	56	-	-	2.49 ± 0.7 ^c^	3.56 ± 0.6 ^c^	-	530 ± 5 ^c^ mg/L	-	7.9 ± 1.8 ^c^	-	-	-	-
De Palma et al., 2012 *Italy* [8]	Electric steel foundry (ICP-MS and AAS)	339	-	0.44 (0.06–1.80) ^a^	-	-	0.90 (0.10–3.39) ^a^	-	-	6.40 (0.50–16.08) ^a,^ (inorganic As)	0.28 (0.13–0.83) ^a^	-	-	-
Soleo et al., 2012 *Italy* [6]	Integrated-cycle steel foundry (ICP-MS and AAS)	49	-	0.10 < 0.10–0.40) ^d^	0.40 (0.00–1.80) ^d^	0.50 (0.08–1.20) ^d^	0.60 (0.20–3.00) ^d^	16.0 (3.3–51.0) ^d^	352 (67.0–2626.0) ^d^	5.0 (0.5–75.0) ^d,^ (inorganic As)	0.40 (<0.006–1.40) ^d^	2.60 (0.20–12.00) ^d^	-	1.50 (0.20–9.20) ^d^
Ściskalska et al., 2014 *Poland* [9]	Copper foundry (AAS)	352	-							S ^a^: 12.76 ^e^*NS ^a^:13.0 ^e^* (inorganic As)	S ^a^: 0.83 ^e^*NS^a^: 0.56 ^e^*			
dos Santos et al., 2015 *Brasil* [7]	Nonferrous metal foundries (GFAAS)	178	-	-	0.64 (5.64) ^f^	-	1.8 (3.15) ^f^	-	-	-	1.27 (4.39)^f^	-	-	-
Wang et al., 2019 *China* [13]	Steel smelting plant (AAS)	162	-	-	-	-	1.18 * (0.005–4.40) ^d^	6.44 * (0.0045–17.80) ^d^			0.93 * (0.17–3.15) ^d^			0.025 * (0.025–1.84) ^d^

N = number of samples; ICP-MS: Inductively coupled plasma-mass spectrometer; GF-AAS: Graphite furnace atomic absorption spectroscopy; S = smokers; NS = nonsmokers; ^a^ = median (5th–95th); ^b^ = mean (range); ^c^ = mean ± DS; ^d^ = median (range); ^e^ = mean; ^f^ = geometric mean (GSD); * µg/g creatinine.
